# Clustering Analysis of Aging Diseases and Chronic Habits With Multivariate Time Series Electrocardiogram and Medical Records

**DOI:** 10.3389/fnagi.2020.00095

**Published:** 2020-05-05

**Authors:** Kuo-Kun Tseng, Jiaqian Li, Yih-Jing Tang, Ching-Wen Yang, Fang-Ying Lin, Zhaowen Zhao

**Affiliations:** ^1^School of Computer Science and Technology, Harbin Institute of Technology (Shenzhen), Shenzhen, China; ^2^Department of Family Medicine, Taichung Veterans General Hospital, Taichung, Taiwan; ^3^Center for Geriatrics and Gerontology, Taichung Veterans General Hospital, Taichung, Taiwan; ^4^Computer and Communication Center, Taichung Veterans General Hospital, Taichung, Taiwan

**Keywords:** electrocardiogram, disease analysis, habit analysis, feature extraction, k-means clustering

## Abstract

**Background:**

With recent technology, multivariate time-series electrocardiogram (ECG) analysis has played an important role in diagnosing cardiovascular diseases. However, discovering the association of wide range aging disease and chronic habit with ECG analysis still has room to be explored. This article mainly analyzes the possible relationship between common aging diseases or chorionic habits of medical record and ECG, such as diabetes, obesity, and hypertension, or the habit of smoking.

**Methods:**

In the research, we first conducted different ECG features, such as those of reduced binary pattern, waveform, and wavelet and then performed a k-means clustering analysis on the correlation between ECGs and the aforementioned diseases and habits, from which it is expected to find a firm association between them and the best characteristics that can be used for future research.

**Results:**

In summary, we discovered a weak and strong evidence between ECG and medical records. For strong evidence, most patients with diabetes are always assigned into a specified group no matter the number of classes in the k-means clustering, which means we can find their association between them. For weak evidence, smokers, obesity, and hypertension have less unique ECG feature vector, enabling clustering them into specific groups, so the ECGs might be used to identify smokers, obesity, and hypertension. It is also interesting that we found obesity and hypertension, which are thought to be related to cardiovascular system. However, they are not highly correlated in our clustering analysis, which might indirectly tell us that the impact of obesity and hypertension to our body is various. In addition, the clustering effect of waveform feature is better than the other two methods.

## INTRODUCTION

In modern clinical medicine, electrocardiogram (ECG) technology is a common diagnostic technique for cardiovascular diseases. Electrocardiogram signal is multivariate time-series data to represent the time of electrical change of the voltage variation, which is detected on the skin. Electrocardiogram technology started in 1903, and so it has existed for more than a century. In this century, rapidly developing ECG technology has made a great contribution to health, and it has become an indispensable routine examination technology in clinics.

Electrocardiography plays an important role in the diagnosis of heart diseases, such as heart rate variability, myocardial ischemia, and myocardial infarction. However, pathological ECG has complex causes and variations. There are significant differences among the ECGs of patients with the same kind of pathology, even among ECGs of the same patient. To make an accurate judgment, physicians usually need to have a rich knowledge and clinical experience. If the physicians have been engaged in identifying a large number of graphics, fatigue could easily cause them to make a mistake. Thus, there is a strong demand for automatic ECG diagnosis, which has been intensively concerned and studied for a period of time.

For automatic ECG diagnosis, there has been much discussion among many scholars, from the suppression of noise to the identification of feature points, detection of the characteristic parameters, waveform category judgment, and even to the final diagnosis approach. Even though some automatic ECG analysis programs have been put into clinical applications from the laboratory, many research results have not been widely accepted by clinicians because ECGs are inaccurate and not uniform, changing from time to time. Moreover, automatic ECG analysis is still confined to heart disease, such as arrhythmia and myocardial infarction. However, for other diseases, such as obesity, hypertension, and diabetes, or habits, such as smoking, there is little related research. In the latest survey, there is only a simple study where heart rate and diabetes are investigated (Niu et al., 2018).

However, we suspect ECG can provide more information on aging diseases and chronic habits. This article is dedicated to finding the relationships between ECGs and common diseases and habits using k-means clustering (Alsabti et al., 1997; Qin et al., 2018) based on ECG features [reduced binary pattern (RBP) (Tseng et al., 2015), waveform (Tseng et al., 2018), and wavelet (Saechia et al., 2005; Chan et al., 2008; Chiu et al., 2009)], expecting that we can extract accurate feature information from ECG signals for further research of automatic diagnosis.

In this article, some related studies are introduced in *Related Studies*, and *Methods With Feature Extraction* presents the used feature extraction methods. In *Methods With K-Means Clustering*, we introduce the k-means clustering method for this research; *Experimental Results* and *Discussion* follow.

## Related Studies

### ECG Signal and Noise

**FIGURE 1:**
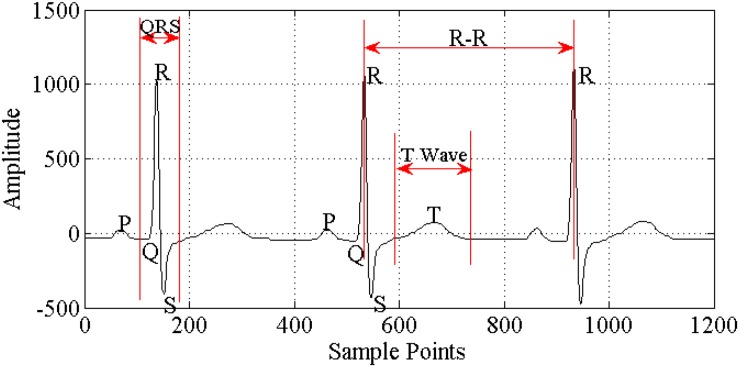
Components of ECG signal.

Since the ECG signal (Figure 1) is relatively weak, it is vulnerable to environmental disturbances. In order to enhance the active ingredients in the ECG signal, suppress noise and interference, and improve the accuracy of waveform detection, not only is it necessary to have high requirements for the anti-interference ability of the ECG recorder hardware, but it is also necessary to have effective ECG signal filtering preprocessing.

Electrocardiogram signal acquisition can be divided into two types: one uses multiple (three or more) ECG leads to record the body surface ECG, so obtaining more comprehensive information about heart activity over a certain period of time and focusing on understanding whether heart electrical activity is abnormal, the nature and degree of any abnormality, and the extent of the lesion area; the other uses one or two ECG leads to record and analyze the ECG continuously for a prolonged period (up to hours or even days), so focusing on understanding the rhythm of the heart’s electrical activity over a long period, whether the individual heart rate is normal, and the time, type, and frequency of arrhythmia occurring. Electrocardiogram disease diagnosis commonly uses the standard 12-lead ECG acquisition method. The ECG should be collected in a relatively quiet environment, with the person relaxed and calm. In general, the basic requirements (Miao et al., 2015) for ECG acquisition include high gain, high common mode rejection ratio, high input impedance, low noise, low drift, high security, and so on.

During the ECG recording, the signal may be corrupted by low-frequency or high-frequency noise, which alters the waveform of the ECG trace from its original structure. To eliminate this noise, the most common types of noise should be considered (Blanco-Velasco et al., 2008; Li et al., 2016) as follows:

*Extraneous noise:* Extraneous noise in the ECG trace may be caused by a variety of noise sources including perspiration, respiration, body movements, and poor electrode contact.

*Electrode motion artifacts:* These are manifested as large-amplitude waveforms primarily caused by stretching that alters the impedance of the skin around the electrode.

*Power line interference:* This is high-frequency noise caused by interference from nearby devices, resulting from improper grounding of the ECG equipment.

*Electromyography noise* (*EMG noise*): EMG noise is caused by the electrical activity of skeletal muscles during periods of contraction or owing to a sudden body movement. Although the frequency component of EMG overlaps considerably with that of the QRS complex, it also extends into higher frequencies.

*Baseline wander:* Baseline wander is caused by body tiny movements and poor electrode contact, 0.05 to 2.00 Hz.

**FIGURE 2:**
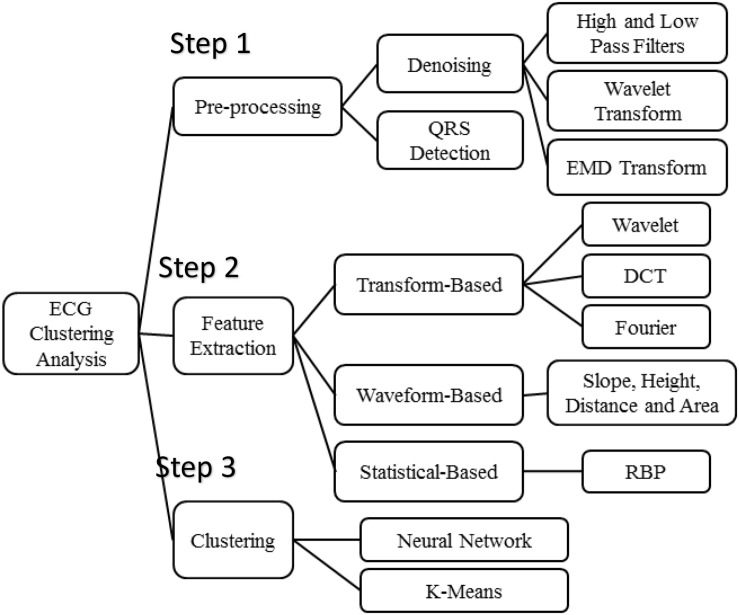
Algorithms of ECG clustering analysis.

There are several commonly used filtering methods (Figure 2), such as the traditional finite impulse response (FIR) filter method (Gholam-Hosseini et al., 1998; Lu et al., 2009; Li et al., 2016), wavelet transform method (Agante and Marques de Sa, 1999; Alfaouri and Daqrouq, 2008), and empirical mode decomposition (EMD) method (Pan et al., 2007; Blanco-Velasco et al., 2008). In recent years, wavelet analysis has been introduced into the ECG filtering process and is widely used. Wavelet transform has good time-frequency localization characteristics, achieving conversion of the signal from time domain to frequency domain. Through multiscale analysis, we can obtain different local characteristics of signals in different scales, which is applicable in analyzing and processing such biomedical signals as ECGs with strong randomness, lower signal-to-noise ratio, non-linear and non-stationary nature, and more singular points.

### Algorithms of ECG Clustering Analysis

Figure 2 presents the three steps of algorithms used in the ECG clustering analysis. According to experimental flow, it consists of three parts sequentially: step 1: preprocessing, step 2: feature extraction, and step 3: clustering. Denoising methods and QRS detection are the main schemes employed in signal preprocessing. The high- and low-pass filters, wavelet transform, and EMD transform are commonly used for ECG denoising.

Most ECG-based disease analyses rely on features that are derived from the ECG signals. According to our survey, the features are usually extracted according to three various models, that is, transform-based, waveform-based, and statistical-based as is shown in Figure 2.

First, transform-based algorithms consist of wavelet transforms (Saechia et al., 2005; Chan et al., 2008; Chiu et al., 2009) and frequency domain transforms, including Fourier transform or discrete cosine transform (Sorwar et al., 2001). Because the wavelet transform contains information in the time and frequency domains, it is more popular than the frequency domain transform.

Waveform-based algorithms extract different time-domain characteristics (distance, height, and area) from fiducial points inside the ECG waveform. These waveform descriptors will be used to match or classify ECG signals in the disease analysis process. These algorithms usually have good accuracy in recognizing regular ECG signals but show the opposite results for irregular data.

An ECG signal can be described as a non-stationary time series that presents some irregularities in the waveform. Unlike the waveform-based algorithms, the transform-based algorithms analyze the non-stationary information based on the signal’s presentation in the frequency domain. Not only is this process slow, but it is also difficult to extract good features for the purpose of classification.

Statistical-based algorithms usually depend on statistical evaluations (count, mean, and variance). They are usually less time-consuming but definitely need a well-designed statistical model to ensure high-quality accuracy. A method based on rank-order statistics was presented to analyze the human heartbeat (Tseng et al., 2015).

For clustering analysis, k-means and neural network are two commonly used clustering methods, but for the sake of simplicity and processing speed, k-means is more applicable in this experiment. Moreover, experiments showed that k-means clustering achieves a positive performance in exploring the relationship between ECGs and the aforementioned diseases and habits.

## Preprocessing

During the ECG recording, the signal may be corrupted by low- or high-frequency noise, which alters the waveform of the ECG trace from its original structure. As mentioned above, the raw data in the Physikalisch-Technische Bundesanstalt (PTB) Diagnostic ECG Database are severely affected by noise, whereas the quality of signal in the ECG database from the local hospital is significantly better that in the PTB database, which influences the preprocessing methods, and QRS detection has impact on feature extraction and even the final clustering of ECG features. Therefore, we may need to investigate different features for different ECG databases in order to find the best features for each diagnostic system.

### ECG Denoising

Extraneous noise in the ECG trace may be caused by a variety of noise sources including perspiration, respiration, body movements, and poor electrode contact. Electrode motion artifacts manifest as large-amplitude waveforms primarily caused by stretching, which alters the impedance of the skin around the electrode. Power line interference is high-frequency noise caused by interference from nearby devices resulting from improper grounding of the ECG equipment. Electromyography noise is caused by the electrical activity of skeletal muscles during periods of contraction or resulting from a sudden body movement. Although the frequency component of EMG overlaps considerably with that of the QRS complex, it also extends into higher frequencies. Commonly used filtering methods include the traditional FIR filter method, wavelet transform method, and the EEMD (ensemble EMD) method.

### QRS Detection

For the waveform feature extraction, a more accurate ECG segmentation is needed, so QRS detection and ECG cutting are needed first. QRS detection is based on the matched filter. In order to improve the accuracy of QRS detection under the conditions of Gaussian noise and variable QRS amplitude, the first-order derivative is used for zero threshold detection. In addition, the two non-linear circuits cut off low-amplitude noise and all spikes that occur within a certain time after QRS detection.

## Methods With Feature Extraction

### RBP Feature Extraction

The idea of RBP algorithm (Tseng et al., 2015) for ECG disease is related to Yang et al. (2003) and Kumar et al. (2005) works, but we expand it to a different field of application.

All ECG signals are non-stationary. Consider an ECG signal as*x* = {*x*_1_,*x*_2_,*x*_3_,…,*x*_N_}, where the real-valued *x*_i_ corresponds to the *i*^th^ input datum. Each pair of consecutive input signals is compared, and the data are categorized into one of the two cases: a decrease or increase in*x*_*i*_. A preliminary reduced function then maps these two cases to 0 or 1, respectively, according to the rule:

$$y_{i}=\left\{ \begin{array}{c} 0,x_{i+1}\leq x_{i}\\ 1,x_{i+1}>x_{i} \end{array}\right.\;\;\left(1\right)$$

**FIGURE 3:**
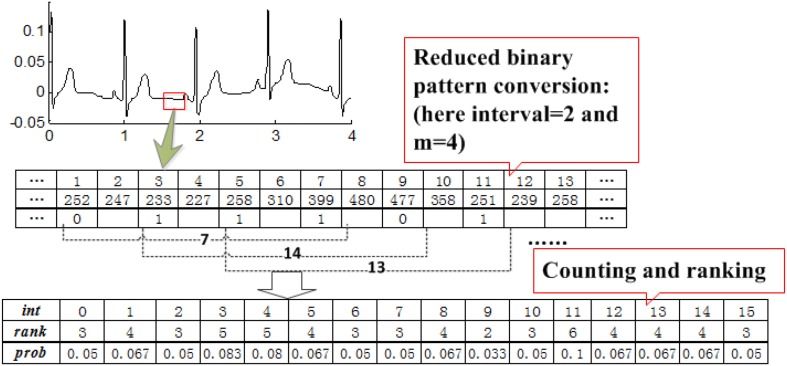
Process of RBP feature extraction.

This procedure converts the ECG signal of length *N* to a binary sequence*Y* = {*y*_1_, *y*_2_,…,*y*_*N*−1_} of length*N*−1. Every *m* bit in *Y* is grouped to construct a reduced binary sequence of length*m*, referred to as an m-bit word, and then all such words are collected to form an RBP *B* = {*b*_1_, *b*_2_,…,*b*_N−m_} where *b_k_* = {*y*_k_, *y*_k + 1_, …,*y*_*k* + *m*−1_}. We then convert each m-bit word*b*_k_ to its decimal expansion *w_k_*. The relative occurrence frequency of *w*_k_ is considered as the features of ECG signals, which we use as the input of clustering method. Figure 3 presents the process of RBP feature extraction.

### Wavelet Feature Extraction

**FIGURE 4:**
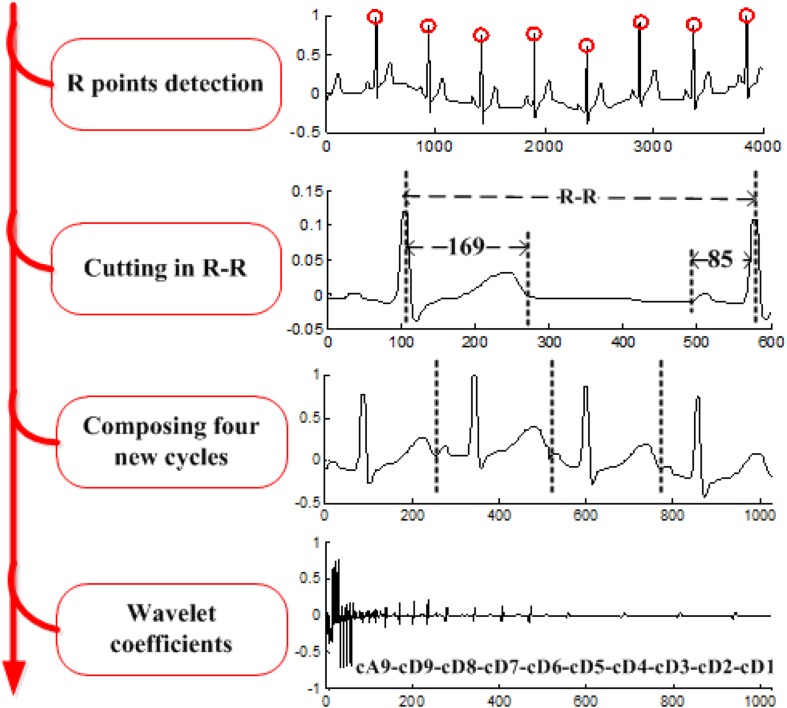
Process of wavelet feature extraction.

The procedures of the wavelet-based algorithm (Chiu et al., 2009) include the following: each R-R cardiac cycle is obtained through R-R detection; the first 169 and the last 85 points in each R-R cycle are assembled to form a 256-point segment; every four segments are grouped, and an n-level discrete wavelet transform (DWT) is performed to obtain the corresponding wavelet coefficients. Four of the computed wavelet coefficients are gathered as a wavelet vector. Here we use the coefficients vector of DWT as the ECG feature. An example with *n* = 9 is illustrated in Figure 4.

### Waveform Feature Extraction

**FIGURE 5:**
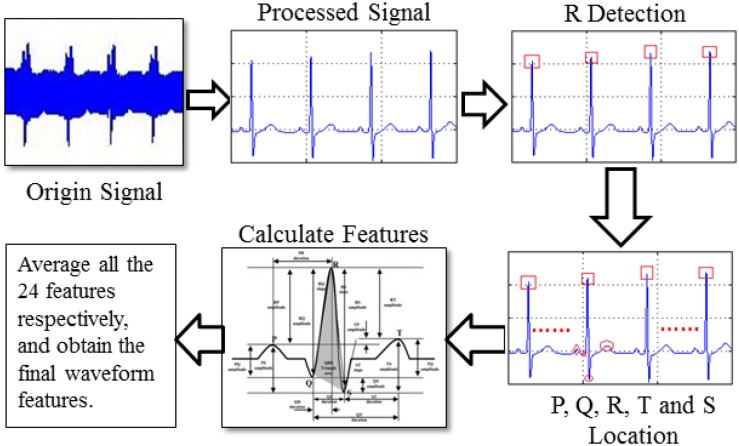
Process of waveform feature extraction.

In a waveform-based feature study (Tseng et al., 2018), a total of 24 features are extracted from five classes: position (P, Q, R, S, T), amplitude (PQ, RQ, TQ, RT, PS, RP, TS, RS, PT, QS), duration (QS, PR, QR, ST, QT), slope (RS, ST and QR), and area (area of the QRS triangle). These features form a feature vectorS. Before extracting the features mentioned above, we need to first accurately locate R, P, Q, T, and S points, which is a key issue for waveform feature extraction. Figure 5 displays the extraction procedure of waveform feature.

## Methods With K-Means Clustering

K-means clustering (Alsabti et al., 1997; Qin et al., 2018) is a rather simple but well-known algorithm for grouping objects. Each object can be represented by a feature vector. First, it extracts ECG features with RBP, waveform, and wavelet and then randomly chooses *k* samples in that vector space, which serve as the initial centers of the clusters. Afterward, all objects are each assigned to their closest center. Usually, the distance measure is chosen by the user and determined by the learning task. In this experiment, we chose the Euclidean distance as follows:

$$D={\left(X_{i}-X_{j}\right)}^{T}\,\left(X_{i}-X_{j}\right)=\sum_{k=1}^{n}{\left(x_{i\,k}-x_{j\,k}\right)}^{2}\;\left(2\right)$$

where *X*_i_ and *X*_j_ are samples, and *x*_ik_ and *x*_jk_ are the k^th^ attribute of *X*_i_ and   *X*_j_.

Then, for each class, a new center is computed by averaging the feature vectors of all objects belonging to this class. The process of assigning objects and recomputing the centers is repeated until the process converges. The algorithm can be proven to converge after a finite number of iterations. Usually, to determine whether the process converges, we take the square error criterion as follows:

$$E=\sum_{i=1}^{k}\sum_{p\in C_{i}}{\left|p-m_{i}\right|}^{2}\;\left(3\right)$$

where *E* is the sum of the square errors of all samples in the database, *P* denotes a sample in the database, and *m*_*i*_ denotes the means of clustering group *C_*i*_.*

## Experimental Results

### Dataset

We conducted a comprehensive experiment on a public ECG dataset, the PTB Diagnostic ECG Database (Bousseljot et al., 1995). The database contains 549 records from 290 subjects (aged 17–87 years, mean 57.2 years; 209 men, mean age 55.5; and 81 women, mean age 61.6; ages were not recorded for 1 female and 14 male subjects, the data of patients 124, 132, 134, and 161 were missed in the PTB database). Each subject is represented by one to five records. Each record includes 15 simultaneously measured signals: the conventional 12 leads (i, ii, iii, avr, avl, avf, v1, v2, v3, v4, v5, v6) together with the three Frank lead ECGs (vx, vy, vz). Each signal is digitized at 1,000 samples per second, with 16-bit resolution over a range of ±16.384 mV. By special request to the contributors of the database, recordings may be available at sampling rates of up to 10 kHz.

Within the header (.hea) file of most of these ECG records is a detailed clinical summary, including age, gender, diagnosis, and, where applicable, data on medical history, medication and interventions, coronary artery pathology, ventriculography, echocardiography, and hemodynamics. The clinical summary is not available for 22 subjects. The diagnostic classes of the remaining 268 subjects are summarized in Table 1.

**TABLE 1:** Demographic of PTB database.

**Diagnostic class**	**All patients**
Age range	17–87
Man mean age	57.2
Man	209
Woman mean age	55.5
Women	81
Myocardial infarction	148
Cardiomyopathy/heart failure	18
Bundle-branch block	15
Dysrhythmia	14
Healthy controls	52
Arterial hypertension	63
Diabetes mellitus	29
Obesity	20
Smoker	73
Not available	22

### Metrics

In the *k* clustering algorithm, two metrics are used to evaluate the relationship between ECGs and diseases or habits.

#### Ratio

This is a commonly used measurement criterion in the clustering analysis. In order to measure the extent of relationship between ECGs and specified disease or habit, the ratio of the number of subjects with disease or habit to the number of subjects in a group is a good criterion. If the ratio in a group is greater than 50%, there should be a significant correlation between ECGs and disease or habit.

#### Concentricity

Sometimes, because of various disturbances, such as shortage of data, it is difficult to achieve a ratio greater than 50%, which cannot indicate there is no correlation between disease and ECGs, so we need a new criterion to measure the performance of clustering. If all or most of samples with the same disease or habit are assigned into the same group, this disease or habit may be identified based on the ECG feature of this group. This is called concentricity.

### Experiment Software

This research mainly focuses on the ECG signal processing; there are many ECG analysis software developed by MATLAB (Natick, MA, United States). Thus, this research uses MATLAB tools as programming language, especially in signal processing, pattern recognition, mathematical modeling, and other aspects. MATLAB also includes matrix arithmetic operation, relational operation, logical operation, conditional operation, and assignment operation, and these matrix operation methods can be copied into array operation. MATLAB does not need to define dimension in matrix operation and has rich matrix function library for various matrix operations.

### Result Based on RBP Feature

In the experiment, we would cluster the PTB database based on RBP features and chose *k* = 24, and 8 (*k* being the number of class), which is helpful in observing the variance of clustering performance with increasing *k*.

In the clustering with the RBP feature as shown in Table 2 with *k* = 4 and 8, typically the ratios in the different classes are relatively less than 50%, which indicates that the samples scatter into different classes with *k* increasing, and the differences among these classes are not obvious. While *k* = 2, the obvious characteristic is that although the number of two groups is approximate, most samples (72.41%) with diabetes are assigned into the second class. Moreover, even though the number of classes in the k-means clustering increases from 2 to 4, even to 8, and the dispersivity of the samples expands, there is always a group in each clustering, for example, group 1 with *k* = 4 and group 4 with *k* = 8, in which the number of samples with diabetes is significantly larger than that in other groups in the same clustering. It demonstrates that diabetes mellitus may be closely related with RBP features of group 2 with *k* = 2, group 1 with *k* = 4 and group 4 with *k* = 8.

**TABLE 2:** Clustering statistics based on RBP feature, *k* = 2, 4, and 8.

***k***	**Category**	**Number**	**Obesity**	**Smoker**	**Hypertension**	**Diabetes**
	Group 1	140	10	30	27	8
2	Group 2	150	10	43	36	21
	Group1	72	9	21	25	14
	Group2	67	1	21	10	6
4	Group3	85	6	16	15	3
	Group4	66	4	15	13	6
	Group 1	42	3	10	10	3
	Group 2	36	2	5	4	3
	Group 3	29	1	8	3	3
	Group 4	53	6	13	19	12
8	Group 5	4	0	1	1	1
	Group 6	6	2	3	2	1
	Group 7	73	5	16	14	3
	Group 8	47	1	17	10	3

For other three research targets, there are no highlighted groups in Table 2 in which samples with same disease or habit present the distribution of aggregation, indicating that we cannot detect the relationships between ECGs and obesity, smoking, and hypertension through RBP feature.

### Result Based on Waveform Feature

Similar to the clustering with RBP feature, we would utilize k-means clustering to cluster the PTB database based on waveform feature. Table 3 displays the results of clustering when the number of groups *k* is 2, 4, and 8.

**TABLE 3:** Clustering statistics based on waveform feature.

***k***	**Category**	**Number**	**Obesity**	**Smoker**	**Hypertension**	**Diabetes**
	Group 1	226	13	37	43	23
2	Group 2	64	7	36	20	6
	Group1	64	7	36	20	6
	Group2	69	1	1	7	1
4	Group3	7	1	1	2	0
	Group4	150	11	35	34	22
	Group 1	21	3	11	6	3
	Group 2	69	0	0	7	1
	Group 3	19	1	9	7	2
	Group 4	9	1	7	3	1
8	Group 5	7	1	1	2	0
	Group 6	149	11	35	34	22
	Group 7	3	2	2	2	0
	Group 8	13	1	8	2	0

With *k* = 4 and 8, most of samples with diabetes are assigned into a specified group, for example, 22 samples in group 4 of *k* = *4* and 22 samples in group 6 of *k* = 8; thus, although the number of groups increases from 4 to 8, this group (group 4 with *k* = 4 and group 6 with *k* = 8) still retains the distribution and composition of various diseases and habits. Moreover, 75.85% of samples with diabetes are allocated in the group with half of all samples, whereas it is a natural and proportional distribution for other three research aspects that only approximately half of their samples are respectively, assigned in this group with half of all samples in the PTB database. It is indicated that diabetes is closely associated with waveform feature extracted from ECGs of the sixth group with *k* = 8.

It is observed that there are four classes with *k* = 4 (corresponding to *k* = 8) with rather higher ratios of smokers, that is, 52.38% in the first group, 77.78% in the fourth group, 66.67% in the seventh group, and 61.45% in the last group. However, there are only three samples in the seventh group, and two of them are also, respectively, patients with obesity or hypertension, which indicates that this group is not practicable and useful because of sparse samples. Perhaps, the sparse samples in the fourth, seventh, and last groups, although with higher ratio of smokers, cannot support the existence of relationship between ECGs and smoking. If we combine these groups with the first group with 21 samples to compose a new group, the problem of sparse samples will be solved, and it is sufficient to determine the relationship between waveform feature of ECGs and smoking, which paves the way of further research about smoking classification.

### Result Based on Wavelet Feature

In the wavelet feature extraction, four segments are grouped, and an n-level DWT is performed to obtain the corresponding wavelet coefficients. Table 4 presents the clustering results with different number of groups *k*.

**TABLE 4:** Clustering statistics based on wavelet feature, *k* = 2, 4, and 8.

***k***	**Category**	**Number**	**Obesity**	**Smoker**	**Hypertension**	**Diabetes**
2	Group 1	257	18	64	54	28
	Group 2	33	2	9	9	1
4	Group1	23	2	9	6	1
	Group2	96	6	27	21	9
	Group3	15	1	8	3	1
	Group4	156	11	29	33	18
8	Group 1	18	0	7	6	0
	Group 2	2	0	0	0	0
	Group 3	58	4	11	14	7
	Group 4	19	1	8	5	1
	Group 5	8	1	4	2	1
	Group 6	105	8	21	21	12
	Group 7	73	5	21	13	8
	Group 8	7	1	1	2	0

Compared with RBP feature and waveform feature, clustering upon wavelet feature cannot achieve a positive performance both in the ratio criterion and concentricity criterion. With the number of groups increasing in the k-means clustering, the samples with specified disease or habit scatter in these groups randomly without any concentricity. Moreover, when the number of groups *k* is 8, the ratios in the groups with enough samples are almost less than 50%. So we cannot find useful information to support the relationship between ECGs and the aforementioned diseases or habits in the k-means clustering with wavelet feature.

## Discussion

In this section, we discuss some issues that may be interest to readers. There are the issues of *k* parameter, feature extraction, ECG dataset, and evidence.

### Issues of k Parameter

#### Why Were Multiple Feature Extraction Algorithms Used?

**FIGURE 6:**
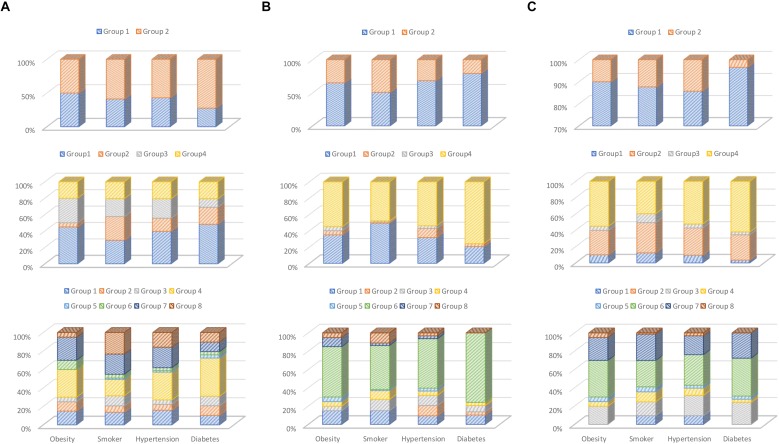
Percentage chart of clustering result: **(A)** RBP-based, **(B)** waveform-based, and **(C)** wavelet-based feature extractions.

Each feature extraction algorithm has different characteristics; for example, in Figure 6, wavelet has a good clustering performance at *k* = 2, but waveform performs better than the other two algorithms at *k* = 4 and *k* = 8.

#### Why Was the Elbow Method Not Used?

We are exploring the effects of different *k* parameters and different feature extraction, so we did not use the elbow method. The reason that we use different *k* values as *k* = 2, 4, and 8 for comparative study, in addition to see the result under different *k*, if a disease can show a better clustering effect under different *k* parameters, it means that it has a higher degree of stability. In the future, if we want design the classification system, we should use the elbow method.

### Issues of Feature Extraction

#### Why Choose These Three Feature Extraction Algorithms for Comparison?

Each feature extraction algorithm has different characteristics; for example, in Figure 6, wavelet has a good clustering performance at *k* = 2, RBP performance after *k* = 2, better than waveform. But waveform performs better than the other two algorithms at *k* = 4 and 8. In addition, the three feature extraction methods we selected are also representative. The RBP feature is statistical method; waveform feature is morphological method, and wavelet is transformation method.

#### Why Not Perform Fusion Clustering on the Three Methods of Feature Extraction?

According to our previous clustering and classification experiments, if multiple feature extractions are used together, the performance is often worse, because different features may interfere with each other. Moreover, this study mainly focuses on the clustering correlation study between ECG and disease; using different features for comparative analysis is more desired in this article. In the future, when we develop the classification and diagnosis system, we will seek more sophisticated feature extraction method; the feature fusion issue will be studied as well.

### Issues of ECG Dataset

#### Why Did We Select Only Aging Chronic Diseases for Clustering Analysis?

We have performed only clustering analysis on arterial hypertension, diabetes mellitus, obesity, and smoker. Because other common heart diseases such as myocardial infarction, cardiomyopathy, bundle-branch block, and dysrhythmia have been proven to be observed by ECG, we do not have motivation to conduct further clustering studies. And the purpose of this article is also to explore the future possibility of automatic ECG diagnosis for chronic diseases.

#### Is the Number of Samples Sufficient?

Both clustering and classification problems use the same preprocessing and feature processing method. If stable clustering results can be obtained, the sample base should be sufficient as in classification and clustering problems. In addition, we have conducted the experiment on a public ECG dataset, the PTB Diagnostic ECG Database. This database has been used for many researches; thus, the clustering results should be credible.

### Issues of Evidence

#### Overall Observation Evidence

As shown in Figure 6, we divided different *k* parameters into *k* = 2, 4, and 8 groups and compared them. At the same time, three different characteristics were compared with the same k-parameter experiments. In addition, in Figure 6, if a group occupies a larger proportion, it indicates that the clustering effect is better. Based on this, our observations are summarized as follows: (Niu et al., 2018) diabetes has strong association with ECG feature; it can almost get better clustering results in different feature extractions and *k* parameters, and other chronic diseases have better clustering performance in experiments with different feature extractions and *k* parameters (Miao et al., 2015). The smokers, obesity, and hypertension have weak association in ECG features; they have less unique ECG feature vector enabling clustering them into specific groups, although they have some correlation in their ECG features (Blanco-Velasco et al., 2008). Waveform feature extractions have better clustering performance than RBP and wavelet.

#### Other Reference for Association Evidence

Previous clinical case studies have explored the relationship between ECG and other chronic diseases. These are mostly the analysis results from physicians’ observation. They found that diabetic patients may have some ECG abnormalities, such as fibrotic changes in the left ventricle (Stern and Sclarowsky, 2009), painless myocardial ischemia (Dweck et al., 2009), and a prolonged QRS duration (Rollins et al., 1992). In addition, as for the other three diseases, we have also found some related studies to prove that they are related to ECG. For example, Renuka Devi et al. (2013) discuss the relationship between heart rate and smoking. The article by Ang and Lang (2008) mentions that left ventricular hypertrophy is related to ECG. The article by Rautaharju et al. (1994) mentions obesity is associated with a wide variety of electrocardiographic abnormalities. Their clinical research and our cluster analysis should prove each other. However, our research results summarize that diabetes has more obvious clustering characteristics than other chronic diseases and should be more suitable for the application of automated ECG diagnosis.

## Conclusion and Future Work

In this article, three different models of feature extraction, RBP-based, wavelet-based, and waveform-based, are investigated and utilized to analyze the possible relationship between ECGs and diseases and habits, such as the aging diseases of diabetes, obesity, and hypertension and the chronic habit of smoking in the k-means clustering. Based on previous experiments and research, our conclusions are summarized as follows:

(1) Waveform feature has better clustering effect in the ECG analysis of the four diseases, and it will be stable under different k parameters. Experiments show that waveform feature is more applicable to the clustering analysis of the correlation in contrast to RBP and wavelet features.
(2) Diabetes has the most obvious clustering effect. The three feature extractions and different k parameters are relatively stable. In the *k*-means clustering based on RBP, waveform, and wavelet features, most patients with diabetes mellitus are always assigned into a specified group no matter the number of groups; that is, samples with diabetes mellitus have an excellent concentricity upon these characteristics extracted from ECGs.
(3) The other three diseases may have less influencing factors, and the clustering characteristics are not significantly stable, which can be further studied in the future. However, the remaining groups with higher ratio of smokers, obesity, and hypertension, the relationship between them and ECGs paves the way of further research about their clustering and classification. The results of smoking, hypertension, and obesity are not positive, and they scatter in every group in the k-means clustering. So, we cannot find strong relationships between ECG and them and cannot choose a better feature to implement the classification and diagnosis of hypertension and obesity based on ECGs.

Regarding future research, we should have the following detailed issues to explore:

(1) The PTB Diagnostic ECG Dataset may not be specialized for discovering the relationship between hypertension, obesity, and diabetes mellitus. Some categories have fewer samples; for example, the number of samples with obesity is 20, which may not perform well for clustering analysis.
(2) The same person may be suffering from a variety of diseases, particularly myocardial infarction, cardiomyopathy, bundle-branch block, and so on, severely affecting the waveform of ECG.
(3) These feature extractions may not include all the useful information for these diseases and habits. Therefore, lack of useful characteristics may bring about clustering performance.
(4) The diseases and habits might have various factors from our body; it is not easy to classify from ECG.

Although our research has explained some question of ECG clustering, there are still some detailed issues that need to be further explored as above. In the future, we will explore these issues and also make further contributions to the future ECG diagnostic system.

## Data Availability Statement

Publicly available datasets were analyzed in this study. This data can be found here: Oeff M., Koch H., Bousseljot R., Kreiseler D. 2012. The PTB diagnostic ECG database. National Metrology Institute of Germany, http://www.physionet.org/physiobank/database/ptbdb.

## Author Contributions

K-KT and JL performed the statistical analysis and participated in design, and drafted the manuscript. Y-JT and C-WY carried out the related studies and participated in collecting data. F-YL and ZZ helped to improve the final manuscript.

## Conflict of Interest

The authors declare that the research was conducted in the absence of any commercial or financial relationships that could be construed as a potential conflict of interest.
